# Effects of dietary traditional Chinese medicine residues on growth performance, intestinal health and gut microbiota compositions in weaned piglets

**DOI:** 10.3389/fcimb.2023.1283789

**Published:** 2023-11-20

**Authors:** Weiguang Sun, Zhong Chen, Zhiyun Huang, Anfeng Wan, Miao Zhou, Jing Gao

**Affiliations:** ^1^ Guangzhou Baiyunshan Xingqun Pharmaceutical Co., Ltd., Guangzhou, China; ^2^ College of Animal Science and Technology, Hunan Agricultural University, Changsha, China; ^3^ Institute of Subtropical Agriculture, Chinese Academy of Sciences, Changsha, China

**Keywords:** traditional Chinese medicine residues, Xiasangju, weaned piglet, weaning stress, gut health, gut microbiota

## Abstract

Weaning stress can induce diarrhea, intestinal damage and flora disorder of piglets, leading to slow growth and even death of piglets. Traditional Chinese medicine residue contains a variety of active ingredients and nutrients, and its resource utilization has always been a headache. Therefore, we aimed to investigate the effects of traditional Chinese medicine residues (Xiasangju, composed of prunellae spica, mulberry leaves, and chrysanthemum indici flos) on growth performance, diarrhea, immune function, and intestinal health in weaned piglets. Forty-eight healthy Duroc× Landrace × Yorkshire castrated males weaned aged 21 days with similar body conditions were randomly divided into 6 groups with eight replicates of one piglet. The control group was fed a basal diet, the antibiotic control group was supplemented with 75 mg/kg chlortetracycline, and the residue treatment groups were supplemented with 0.5%, 1.0%, 2.0% and 4.0% Xiasangju residues. The results showed that dietary Xiasangju residues significantly reduced the average daily feed intake, but reduced the diarrhea score (*P* < 0.05). The 1.0% and 2.0% Xiasangju residues significantly increased the serum IgM content of piglets, and the 0.5%, 1.0%, 2.0% and 4.0% Xiasangju residues significantly increased the serum IgG content, while the 1.0%, 2.0% and 4.0% Xiasangju residues significantly increased the sIgA content of ileal contents (*P* < 0.05). Dietary Xiasangju residues significantly increased the villus height and the number of villus goblet cells in the jejunum and ileum, and significantly decreased the crypt depth (*P*<0.05). The relative mRNA expression of *IL-10* in the ileum was significantly increased in the 1% and 2% Xiasangju residues supplemented groups (*P* < 0.05), while *IL-1β* in the ileum was downregulated (*P* < 0.05). Xiasangju residues improved the gut tight barrier, as evidenced by the enhanced expression of *Occludin* and *ZO-1* in the jejunum and ileum. The diets with 1% Xiasangju residues significantly increased the relative abundance of *Lactobacillus johnsonii*, and 2% and 4% Xiasangju residues significantly increased the relative abundance of *Weissella jogaeotgali* (*P* < 0.05). Dietary supplementation with 0.5%, 1.0%, 2% and 4% with Xiasangju residues significantly decreased the relative abundance of *Escherichia coli* and *Treponema porcinum* (*P* < 0.05). In summary, dietary supplementation with Xiasangju residues improves intestinal health and gut microbiota in weaned piglets.

## 1 Introduction

With the increasing scale and intensification of pig production, early weaning of piglets has become a major mode to improve production efficiency (Zhao et al., 2019; Ramirez et al., 2022). However, due to the underdeveloped immune function and gastrointestinal digestive function of early weaned piglets, they show high sensitivity to solid feed and pathogenic bacteria, which easily induce weaning stress, further impair gut function and gut microbiota, cause diarrhea, anorexia, listlessness and other symptoms, and reduce production efficiency (Jayaraman and Nyachoti, 2017; Heuß et al., 2019). According to incomplete statistics, the prevalence of diarrhea in early weaned piglets is as high as 20%, resulting in significant economic losses to the pig industry (Hu et al., 2018). ​While antibiotics and other drugs can alleviate the stress of weaning piglets, long-term use of nonstandard medications has caused some harm, such as bacterial resistance, drug residues, and environmental pollution (Tucker et al., 2021). As a result, the livestock industry is under tremendous pressure to ban antibiotics in feed and reduce them in treatment. Therefore, there is an urgent need to develop new strategies to replace drugs such as antibiotics. As a potential green, safe, environmentally friendly and efficient alternative to antibiotics, plant extracts have attracted increasing attention from all sectors of society in feed production and animal husbandry (Zhao et al., 2020; Li et al., 2022; Zhao et al., 2022). ​Plant extracts, including polyphenols, polysaccharides, alkaloids, and other functional components, have been shown to have positive effects on antibacterial, anti-inflammatory, antiviral, antioxidant, growth promotion, immune regulation, and improved gut microbiota in animals (Du et al., 2022; Yu et al., 2022; Zhao et al., 2022).

Xiasangju is a classic traditional Chinese medicine formula that comprises Prunellae spica, mulberry leaves, and Chrysanthemum indici flos. It was first recorded in the “Analysis of Warm Diseases (Wen Bing Tiao Bian)” of the Qing Dynasty approximately 1798 AD (Wu et al., 2022). Xiasangju is known for its ability to liver-clearing, enhance eyesight, heat-clearing, and detoxification. Recent research has revealed that Xiasangju possesses antibacterial, antiviral, antioxidant, antitumor, immunomodulatory, and liver-protective properties (Xia et al., 2016; Huang et al., 2021; Wu et al., 2022). In southern China, Xiasangju has been widely used as a herbal tea formula, and is regularly employed by clinicians to address conditions such as cold, fever, and sore throat (Xia et al., 2017). ​In recent years, a growing number of Xiasangju extract products have been developed, such as Xiasangju granules, Xiasangju capsules, Xiasangju oral liquids and Xiasangju herbal tea drinks. As a result, China has a large amount of Xiasangju residues to dispose of each year.

In industry, traditional Chinese medicine residues are generally treated as waste, which wastes resources and pollents the environment. Chinese herbal medicine is affected by the extraction process, and the residue of medicinal components still contains a large number of nutrients and bioactive substances (Huang et al., 2021; Luo et al., 2023). Previous research has indicated that Xiasangju contains a diverse array of active ingredients (Wu et al., 2022). Specifically, prunella spica is characterized by the presence of key compounds such as rosaminic acid, luteolin, rutin, and quercetin (Bai et al., 2016). Likewise, chlorogenic acid, rutin, and quercetin serve as the principal active components in mulberry leaves (Ma et al., 2022), while chrysanthemum indici flos includes chlorogenic acid, luteolin, and monoglycosides as its major bioactive constituents (Shao et al., 2020). These active substances have been found to exert various biological effects, including promoting animal growth, relieving diarrhea, acting as antioxidants, mitigating stress, improving intestinal flora, and modulating immune responses (Kuralkar and Kuralkar, 2021; Tsiplakou et al., 2021). Therefore, the application of Chinese medicine residue to animal production is a measure worth promoting, and it is also an important part of the recycling of Chinese medicine residue resources.

Although existing studies support the potential of Xiasangju to ameliorate weaning stress in piglets (Naveed et al., 2018; Canibe et al., 2022; Gao et al., 2022), there is a lack of information on the effects of Xiasangju residues on weaning stress. Therefore, in this study, we evaluated the effects of different levels of Xiasangju residues on the health and gut microbiota of weaned piglets, hoping to provide a new strategy for alleviating weaning stress in piglets, promote the development and utilization of Xiasangju residues as feed, and alleviate environmental stress.

## 2 Materials and methods

This animal study was reviewed and approved by the Hunan Agricultural University Institutional Animal Care and Use Committee (202105). Written informed consent was obtained from the owners for the participation of their animals in this study.

### 2.1 Materials

Dried Xiasangju residue (moisture content 6.64 ± 0.34%) was provided by Guangzhou Baiyunshan Xingqun Pharmaceutical Co., LTD. The preparation of the residue was as follows: Prunella, mulberry leaves, and wild chrysanthemums were sampled according to a weight ratio of 500:175:80, and 10 times the water was added. The traditional Chinese medicine was decoction at 100°C for 1.5 hours, 2 times in total, and filtered and dried to obtain the residue of Xiasangju.

### 2.2 Quantitative analysis of active ingredients in residues

To determine the content of active ingredients in residues, the LC−MS method was selected for quantitative analysis. Five milligrams of linoside, romaricinic acid, isobromaricin, acacetin, caffeic acid, chlorogenic acid, neochlorogenic acid, cryptochlorogenic acid, isochlorogenic acid A, isochlorogenic acid B, and isochlorogenic acid C were placed in a 25 mL volumetric flask, and a small amount of methanol was added to dissolve, returned to room temperature, and then volume to the scale line with methanol. A single standard stock solution of 0.2 mg/mL was prepared. More than 200 μL of a single standard stock solution was placed in a 10 mL brown volumetric flask, and methanol was used to make a 4 μg/mL mixed standard stock liquor (the concentration of each component was 4 μg/mL). Then, the above control mixed standard stock solution was diluted to make mixed standard working curve correction solutions of 2 μg/mL, 1 μg/mL, 0.5 μg/mL, 0.2 μg/mL, and 0.1 μg/mL. A linear equation was drawn with the concentration of active ingredients as the abscissa and the peak area of EIC as the ordinate. The concentration of active ingredients in the tested samples was calculated by the standard curve equation. The specific detection parameters are shown in **Table 1**.

**Table 1:** Quantitative analysis parameters of active components of residue by LC-MS.

Item	Method description
The chromatographic conditions for LC analysis:	
Chromatographic column	Agilent Eclipse XDB-C18 (2.1 mm × 150 mm, 1.8 μm)
Mobile phase:	
Aqueous phase (A)	0.1% formic acid in water
Organic phase (B)	Acetonitrile
Gradient elution program:	
0-8 min:	2%-30% B
8-25 min:	30%-95% B
25-30 min:	95% B
30.1 to 35 min:	2% B
Column temperature:	35°C
Autosampler temperature:	Room temperature (25 ± 2°C)
Flow rate:	0.3 mL/min
Injection volume:	1 μL
DAD (Diode Array Detector) collected data using full-wavelength scanning mode with a scanning range of 210-400 nm.	
The high-resolution primary mass spectrometry (HRMS) conditions:	
Ion source:	Agilent Dual AJS ESI source
Scan mode:	Positive
Full sweep range:	m/z 100-1000
Sheath temperature:	350 °C
Sheath gas flow rate:	11.0 L/min
Drying temperature:	345°C
Drying gas pressure:	45 psi
Drying gas flow rate:	10 L/min
Capillary voltage:	4000 V
Fragmentor:	135 V
Data acquisition software:	Agilent MassHunter Acquisition Workstation Software (B.08.00)

For high-resolution secondary mass spectrometry in target MS/MS mode, the ion source parameters were the same as the primary mass spectrometry conditions. The appropriate collision energy was chosen based on the molecular weight and structural stability of the target compound, within the range of 5 to 45 eV.

### 2.3 Animals and dietary treatments

Forty-eight healthy Duroc× Landrace × Yorkshire castrated male weanling piglets aged 21 days with similar body conditions were randomly divided into 6 groups, each group of eight repeats each repeat a piglet. The experiment began the next day and lasted for 21 days. The control group was fed a basal diet, the antibiotic control group received 75 mg/kg chlortetracycline (chlortetracycline premix purchased from Zhumadian Huazhong Zhengda Co., LTD.), and the residue treatment groups were supplemented with 0.5%, 1.0%, 2.0% and 4.0% Xiasangju residues. The residues were crushed through a 60-mesh sieve, the basic diet (**Table 2**) was prepared according to NRC2012, and the nutritional level met the NRC recommendation.

**Table 2:** Ingredient composition and nutrient level of the basal diet (%, as-fed basis).

Ingredients	Content	Nutrient level*^b^*	Content
Corn	55.00	Digestible energy, kcal/kg	3542.1
Fermented soybean meal	31.00	Crude protein	18.51
Soybean oil	3.00	Calcium	0.80
Glucose	2.00	Total phosphorus	0.60
Sucrose	3.00	Available phosphorus	0.38
Limestone	1.00	SID Lysine	0.1.37
CaHPO_4_	1.50	SID Methionine	0.53
NaCl	0.30	SID Methionine + cystine	0.0.73
Citric acid	0.90	SID Threonine	0.77
Choline chloride	0.10	SID Tryptophan	0.19
Lysine	0.60		
Methionine	0.30		
Threonine	0.30		
Premix*^a^*	1.00		
Total	100.00		

^a^Premix provided Cu 126.00 mg/kg diet; Fe 102.00 mg; Zn 106.50 mg; Mn 17.70 mg; I 0.18 mg; Se 0.14 mg; VA 8000U; VB_1_ 1.8 mg; VB_2_ 4.4 mg; VB_6_ 4.4 mg; VB_12_ 0.025 mg; VC 150.00 mg; VD_3_ 1000.00U; 25-OH-D_3_ 0.025 mg; VE 120.00 mg; Pantothenic acid 12.40 mg; Niacinamide 25.00 mg; Folic acid 0.88 mg; Biotin 132.00 mg. ^b^Ileal standard digestible amino acids (SID) were calculated values, and the rest were measured values.

### 2.4 Sample collection

At the end of the experiment, blood samples were taken from the anterior vena cava of all piglets using a 10 ml common blood collection tube, left at room temperature for 20 min, and then centrifuged at 845 rcf (g) for 10 min. The upper serum was collected in sterile frozen tubes, cached in liquid nitrogen, and then stored at -80°C. They were anesthetized with 3% sodium pentobarbital at a dose of 25 mg/kg and killed by exenteration, and the abdominal cavity was opened to separate the viscera and intestine. Two 2 cm pieces of intestinal tissue were taken from the anterior segment of the jejunum and the posterior segment of the ileum, one of which was placed in a 4% paraformaldehyde solution for subsequent histological analysis. Another portion was quickly cleaned with normal saline, placed in a 2 ml sterile cryostat and stored in liquid nitrogen. Approximately 10 cm of the intestinal tract was taken from the anterior segment of the colon, and a small opening was made with a scalpel, after which the ends were bound together with a string. The gut contents were loaded into a 2 ml sterile cryostat and stored in liquid nitrogen. Furthermore, the ileal digesta was collected in a 2 ml sterile cryostat and stored in liquid nitrogen.

### 2.5 Growth performance

Each piglet was weighed on day 1 and day 21, and the amount of food supplied and remaining was recorded daily to calculate the average daily feed intake (ADFI), average daily gain (ADG) and feed-to-weight ratio (F/G). The piglets were scored by observing their diarrhea at approximately 15:00 daily, and the scoring criteria were as follows: 1: solid hard stool; 2: slightly loose stool; 3: soft stool, partially formed; 4: semiliquid stool; and 5: stool-water separated, watery, unformed.

### 2.6 Organ index

After the piglets were slaughtered, the organs were separated, and the surface liquid of the organs was quickly blotted dry with filter paper and then weighed. The formula is as follows: organ index = organ weight (g)/live weight of piglets (kg).

### 2.7 Serum biochemical parameter analysis

The contents of serum total protein, albumin, total bile acid, glucose, triglyceride, urea nitrogen, total cholesterol, high-density lipoprotein cholesterol, low-density lipoprotein cholesterol, alanine aminotransferase, aspartate aminotransferase, alkaline phosphatase, and lactate dehydrogenase were detected by an automatic biochemical analyzer and its matching reagents (KHB 450, Shanghai Kehua bioengineering co., Ltd., Shanghai, China), according to Shi et al. (2022).

### 2.8 Immunoglobulin analysis

Serum IgA, IgG and IgM levels were detected by ELISA kits (Jiangsu Meimian Industrial Co., Ltd., Jiangsu, China), and ileal digesta sIgA content was detected by ELISA kits (Quanzhou Ruixin Biological Technology Co., Ltd., Quanzhou, China). All detection steps were carried out according to the instructions of the kit, according to Yu et al. (2021).

### 2.9 Intestinal morphology

Following our previous study (Wang et al., 2022), intestinal tissue fixed in a 4% paraformaldehyde solution was cut and dehydrated, embedded in paraffin, sectioned, dewaxed, stained with hematoxylin and eosin, dehydrated, and sealed with neutral glue. Images were observed and collected with a microscope imaging system (Carl Zeiss, Germany) to measure intestinal villus height (VH) and crypt depth (CD). The ratio of villus height to crypt depth (V/C) was calculated for five randomly selected fields per slice.

### 2.10 AB-PAS Staining

Following our previous study, tissue paraffin blocks were cut into slices, deparaffinized and rehydrated, stained according to the instructions of the AB-PAS test kit (Nanjing Jiancheng Bioengineering Institute, Nanjing, China), sealed with neutral resin, and finally observed and photographed with a Carl Zeiss Microimaging System. Ten intact villi and crypts from each of the piglet samples were selected for goblet cell number determination, and the result was expressed as the number of goblet cells within each villus.

### 2.11 Quantitative real-time PCR analysis

Intestine tissue was frozen and ground in liquid nitrogen. Total RNA was isolated using RNAiso Plus (TaKaRa, Dalian, China), followed by reverse transcription using the PrimeScript^™^ RT Reagent Kit with gDNA Eraser (TaKaRa, Dalian, China). Fluorescence quantification was then performed with TB Green^®^ Fast qPCR Mix in a LightCycler 480 System II. The primers used in this study were designed based on porcine sequences (**Table 3**); PCR cycles and relative expression assays were performed according to our previous study (Yin et al., 2018).

**Table 3:** Primers used for gene expression analysis by real-time PCR.

Gene	Primer sequence (5′–3′)	Product length, bp
*IL-1β*	F: CCTGGACCTTGGTTCTCT	123
	R: GGATTCTTCATCGGCTTCT	
*IL-10*	F: TCGGCCCAGTGAAGAGTTTC	127
	R: GGAGTTCACGTGCTCCTTGA	
*IL-6*	F: AAATGTCGAGGCCGTGCAGATTAG	86
	R: GGGTGGTGGCTTTGTCTGGATTC	
*TNF-α*	F: ACAGGCCAGCTCCCTCTTAT	102
	R: CCTCGCCCTCCTGAATAAAT	
*ZO-1*	F: TTGATAGTGGCGTTGACA	126
	R: CCTCATCTTCATCATCTTCTAC	
*Claudin-1*	F: GCATCATTTCCTCCCTGTT	97
	R: TCTTGGCTTTGGGTGGTT	
*Occludin*	F: CAGTGGTAACTTGGAGGCGTCTTC	103
	R: CGTCGTGTAGTCTGTCTCGTAATGG	
*Mucin2*	F: CTGTGTGGGGCCTGACAA	65
	R: AGTGCTTGCAGTCGAACTCA	
*β-actin*	F: CTGCGGCATCCACGAAACT	147
	R: AGGGCCGTGATCTCCTTCTG	

IL-1β, interleukin-1β; IL-6, interleukin-6; IL-10, interleukin-10; TNF-α, tumor necrosis factor alpha; ZO-1, tight junction protein 1.

### 2.12 Microbial analysis

Total microbial genomic DNA was extracted from the colonic digesta using the Power Fecal DNA Extraction Kit (MOBIO, USA). Universal primers 341F (5’-ACTCCTACGGGAGGCAGCAG-3’) and 806R (5’-GGACTACHVGGGTWTCTAAT-3’) were used to amplify the V3-V4 region of the 16S rRNA gene. After purification, the PCR product was used to construct the library using the NEB NextB UltraTM DNA Library Prep Kit from New England Biolabs, Inc. ‘s Lumina Library Construction Kit. The constructed library is quantized and probed by a Qubit. After eligibility, Illumina MiSeq PE300 was used for on-machine sequencing. The raw sequences were analyzed using QIIME, version 1.7.0. Initial reads were mass filtered, denoised, and assembled, and chimera sequences were removed according to the method of Deblur et al (Callahan et al., 2016). Only ASVs with at least 2 reads and present in more than 2 samples were kept. PICRUSt2 was used to predict gut microbiota function. All data from the NovoMagic cloud platform (https://magic.novogene.com/) were analyzed.

### 2.13 Statistical analysis

Data on growth performance, organ index, serum parameters, and RT−qPCR were analyzed by one-way ANOVA with SPSS 26.0 statistical software, and multiple comparisons were performed using Duncan’s method. *P* < 0.05 was considered a significant difference, and *P* < 0.01 was considered an extremely significant difference. The results are expressed as the mean ± SD.

## 3 Results

### 3.1 Quantitative analysis of active ingredients in residues

The results of LC-MS quantitative analysis are shown in **Table 4**. The main active ingredients in residues included lineside (83.22 ± 56.2 μg/g), rosmarinic acid (25.92 ± 13.47 μg/g), isrosmarinic acid (10.68 ± 4.71 μg/g), caffeic acid (10.18 ± 5.53 μg/g), and several different types of chlorogenic acid (9.08 ± 4.75 μg/g).

**Table 4:** Quantitative analysis of active ingredients in the residues of Xiasangju.

Item	Neochlorogenic acid	Cryptochlorogenic acid	Chlorogenic acid	Caffeic acid	Isochlorogenic acid B	Isochlorogenic acid A	Isochlorogenic acid C	Rosmarinic acid	Isorosmarin	Linarin	Acacetin
Content	3.86 ± 1.79	3.84 ± 3.01	4.25 ± 2.02	9.08 ± 4.75	1.6 ± 1.01	2.38 ± 1.21	3.98 ± 1.48	25.92 ± 13.47	10.68 ± 4.71	83.22 ± 56.2	10.18 ± 5.53

The mean value (n = 15) was used for the quantitative analysis of active ingredients in the residues of Xiasangju.

### 3.2 Growth performance and diarrhea score

The results of growth performance and diarrhea score are shown in **Table 5**. Compared with the control group, Xiasangju residues had no significant effect on the final test body weight, average daily gain or feed/gain of weanling piglets (*P* > 0.05). Adding 0.5%, 1.0%, and 2.0% Xiasangju residues significantly reduced the diarrhea score of piglets (*P* < 0.05), and the effect was equivalent to chlortetracycline.

**Table 5:** Effect of Xiasangju residues on the growth performance and diarrhea score of weaned piglets.

Item	CON	AUR	X1	X2	X3	X4	P value
Initial body weight(kg)	6.82 ± 0.03	6.83 ± 1.06	6.94 ± 1.07	6.82 ± 0.97	6.77 ± 1.04	6.94 ± 0.91	1.000
Final body weight(kg)	11.46 ± 0.34	11.24 ± 1.36	10.77 ± 1.86	11.00 ± 0.18	11.23 ± 1.20	10.43 ± 0.42	0.932
ADG(kg/d)	0.22 ± 0.01	0.21 ± 0.01	0.19 ± 0.04	0.20 ± 0.04	0.22 ± 0.01	0.17 ± 0.02	0.368
ADFI(kg/d)	0.49 ± 0.02^a^	0.42 ± 0.00^b^	0.40 ± 0.03^b^	0.39 ± 0.03^b^	0.42 ± 0.03^b^	0.41 ± 0.01^b^	0.049
F/G	2.21 ± 0.06	2.01 ± 0.14	2.20 ± 0.29	1.99 ± 0.24	2.00 ± 0.06	2.50 ± 0.25	0.197
Diarrhea score	3.88 ± 0.07^a^	3.14 ± 0.01^cd^	3.23 ± 0.17b^cd^	3.08 ± 0.31^d^	3.50 ± 0.01^bc^	3.56 ± 0.06^ab^	0.011

CON, basal diet fed; AUR, 75 mg/kg chlortetracycline added to the basal diet; X1, 0.5% Xiasangju residues added to the basal diet; X2, 1.0% Xiasangju residues added to the basal diet; X3, 2.0% Xiasangju residues added to the basal diet; X4, 4.0% Xiasangju residues added to the basal die; ADG, average daily gain; ADFI, average daily feed intake; F/G, the ratio of feed intake to gain.

The mean values of the same row of data without the same superscript letter were significantly different at the P-value (n=6).

### 3.3 Organ index

As shown in **Table 6**, Xiasangju residues and chloromycetin had no significant effect on the liver index, kidney index or spleen index of piglets compared with the control group (*P* > 0.05).

**Table 6:** Effect of Xiasangju residues on the organ indices of weaned piglets.

Item	CON	AUR	X1	X2	X3	X4	P value
Liver index	28.25 ± 1.58	25.96 ± 2.08	25.92 ± 2.29	27.49 ± 4.16	25.52 ± 3.53	28.64 ± 3.04	0.187
Renal index	5.83 ± 0.75	5.98 ± 1.09	5.52 ± 0.36	6.08 ± 0.33	6.24 ± 0.68	5.65 ± 1.69	0.717
Spleen index	2.00 ± 0.47	2.30 ± 0.56	2.17 ± 0.35	2.37 ± 0.58	2.12 ± 0.20	2.49 ± 1.39	0.781

CON, basal diet fed; AUR, 75 mg/kg chlortetracycline added to the basal diet; X1, 0.5% Xiasangju residues added to the basal diet; X2, 1.0% Xiasangju residues added to the basal diet; X3, 2.0% Xiasangju residues added to the basal diet; X4, 4.0% Xiasangju residues added to the basal die.

### 3.4 Serum biochemical parameters

**Table 7** shows the effect of the Xiasangju residues on the serum biochemical parameters of piglets. In terms of serum enzyme content, compared with the control group, adding 0.5% and 2.0% Xiasangju residues could significantly reduce the content of AST (*P* < 0.05). Adding 0.5%, 1.0%, 2.0% and 4.0% Xiasangju residues significantly reduced the ALP content (*P* < 0.05), and adding 2.0% Xiasangju residues significantly reduced the LDH content (*P* < 0.05).

**Table 7:** Effect of Xiasangju residues on serum biochemical parameters of weaned piglets.

Item	CON	AUR	X1	X2	X3	X4	*P* value
ALT(U/L)	68.89 ± 13.28	68.52 ± 15.40	55.84 ± 9.73	58.69 ± 16.44	52.79 ± 12.32	65.66 ± 10.10	0.208
AST(U/L)	157.09 ± 51.52^a^	145.20 ± 44.60^ab^	100.14 ± 18.76^bc^	124.66 ± 38.70^abc^	80.09 ± 10.60^c^	121.53 ± 43.49^abc^	0.032
ALP(U/L)	402.95 ± 49.96^a^	305.58 ± 44.09^b^	242.19 ± 34.40^c^	313.63 ± 38.00^b^	294.08 ± 39.65^b^	274.12 ± 26.47^bc^	0.000
LDH(U/L)	815.67 ± 146.01^a^	703.33 ± 133.54^ab^	666.67 ± 170.99^ab^	845.00 ± 197.40^a^	583.17 ± 54.47^b^	730.17 ± 125.71^ab^	0.042
TBA(umol/L)	63.54 ± 13.10	56.09 ± 11.15	60.24 ± 18.36	58.27 ± 20.90	46.60 ± 5.03	71.72 ± 13.58	0.111
TP(g/L)	48.80 ± 6.40^a^	32.86 ± 4.14^b^	58.04 ± 11.12^a^	57.71 ± 11.18^a^	54.01 ± 5.80^a^	54.77 ± 2.65^a^	0.000
ALB(g/L)	9.87 ± 2.43^c^	11.95 ± 1.28^c^	8.23 ± 1.44^c^	27.65 ± 4.68^b^	31.91 ± 4.41^a^	34.48 ± 3.91^a^	0.000
GLB(g/L)	36.86 ± 5.81^b^	17.17 ± 6.40^d^	50.00 ± 10.35^a^	32.50 ± 16.69^bc^	22.33 ± 4.37^cd^	20.50 ± 2.07^d^	0.000
A/G	0.28 ± 0.13^c^	0.46 ± 0.18^c^	0.17 ± 0.04^c^	1.24 ± 0.55^b^	1.57 ± 0.24^a^	1.59 ± 0.28^a^	0.000
GLU(mmol/L)	5.22 ± 0.89^b^	4.97 ± 0.34^b^	5.43 ± 0.33^b^	5.44 ± 0.63^b^	5.28 ± 0.89^b^	6.52 ± 1.08^a^	0.023
BUN(mmol/L)	5.85 ± 1.18^ab^	6.07 ± 0.96^a^	5.71 ± 0.47^abc^	4.68 ± 1.00^cd^	4.55 ± 0.59^d^	4.85 ± 0.91^bcd^	0.015
TC(mmol/L)	2.31 ± 0.27^a^	1.85 ± 0.35^b^	2.33 ± 0.23^a^	2.21 ± 0.18^a^	2.11 ± 0.26^ab^	2.16 ± 0.35^ab^	0.062
TG(mmol/L)	0.85 ± 0.14	0.87 ± 0.33	0.73 ± 0.20	0.81 ± 0.25	0.76 ± 0.26	0.73 ± 0.29	0.874
HDL-C(mmol/L)	0.78 ± 0.09^a^	0.62 ± 0.07^b^	0.81 ± 0.04^a^	0.79 ± 0.03^a^	0.87 ± 0.19^a^	0.81 ± 0.23^a^	0.048
LDL-C(mmol/L)	2.07 ± 0.37^a^	1.34 ± 0.39^b^	2.18 ± 0.44^a^	1.12 ± 0.32^bc^	0.90 ± 0.08^c^	0.93 ± 0.20^c^	0.000

CON, basal diet fed; AUR, 75 mg/kg chlortetracycline added to the basal diet; X1, 0.5% Xiasangju residues added to the basal diet; X2, 1.0% Xiasangju residues added to the basal diet; X3, 2.0% Xiasangju residues added to the basal diet; X4, 4.0% Xiasangju residues added to the basal die; ALT, glutamic-pyruvic transaminase; AST, aspartate aminotransferase; ALP, alkaline phosphatase; LDH, lactic dehydrogenase; TBA, total bile acid; TP, total protein; ALB, albumin; GLB, globulin; A/G, albumin/globulin; GLU, glucose; BNU, blood urea nitrogen; TC, total cholesterol; TG, triglycerides; HDL-C, high-density lipoprotein cholesterol; LDL-C, low-density lipoprotein cholesterol.

The mean values of the same row of data without the same superscript letter were significantly different at the P-value (n=6).

In terms of serum protein content, compared with the control group, the content of albumin was significantly increased by adding 1.0%, 2.0% and 4.0% Xiasangju residues (*P* < 0.05), the content of globulin was significantly increased by adding 0.5% Xiasangju residues (*P* < 0.05), and the content of globulin was significantly decreased by adding 2.0% and 4.0% Xiasangju residues (*P* < 0.05). At the same time, the ratio of albumin to globulin was significantly increased by 1.0%, 2.0% and 4.0% Xiasangju residues (*P* < 0.05).

In terms of glucose and lipid metabolism, supplementation with 2.0% and 4.0% Xiasangju residues significantly reduced serum low-density lipoprotein cholesterol content (*P* < 0.05), while 4.0% Xiasangju residues significantly increased serum glucose content (*P* < 0.05). In addition, the serum urea nitrogen content was significantly reduced by adding 1.0%, 2.0% and 4.0% Xiasangju residue (*P* < 0.05).

### 3.5 Immunoglobulin content

**Figure 1:**
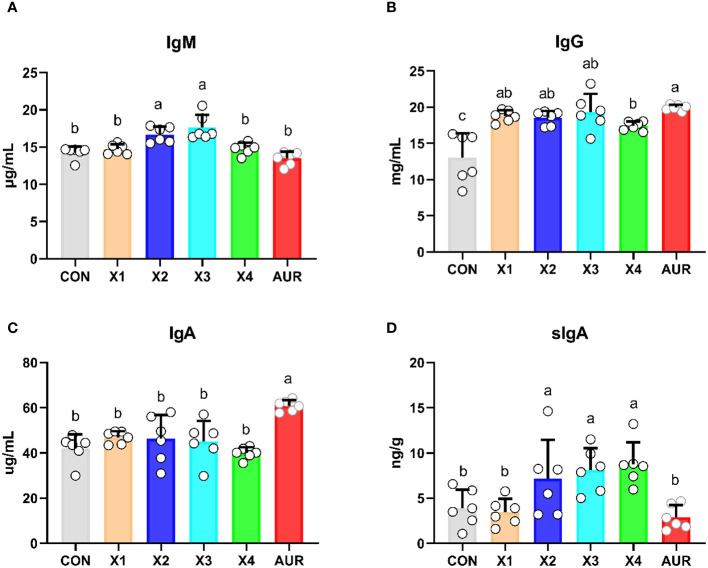
Effect of Xiasangju residues on immunoglobulin content in weaned piglets. **(A)**, serum immunoglobulin A content. **(B)** Serum immunoglobulin G content. **(C)** serum immunoglobulin A content. **(D)** secretory immunoglobulin A content of ileal contents. CON, basal diet fed; AUR, 75 mg/kg chlortetracycline added to the basal diet; X1, 0.5% Xiasangju residues added to the basal diet; X2, 1.0% Xiasangju residues added to the basal diet; X3, 2.0% Xiasangju residues added to the basal diet; X, 4.0% Xiasangju residues added to the basal die; IgM, immunoglobulin M; IgG, immunoglobulin G; IgA, immunoglobulin A; sIgA, secretory immunoglobulin A. Means without the superscript same letter were significantly different at the *P*-value(n=6).

As shown in **Figure 1**, compared with the control group, the addition of 1% and 2% Xiasangju residues significantly increased the serum IgM level (*P* < 0.05), the addition of 0.5%, 1%, 2% and 4% Xiasangju residues all increased the serum IgG level (*P* < 0.05), and the addition of 1%, 2% and 4% Xiasangju residues all increased the level of sIgA in the ileum contents of piglets (*P* < 0.05), while the effect of Xiasangju residues on the serum IgA level was not significant (*P* > 0.05).

### 3.6 Intestinal morphology

**Figure 2:**
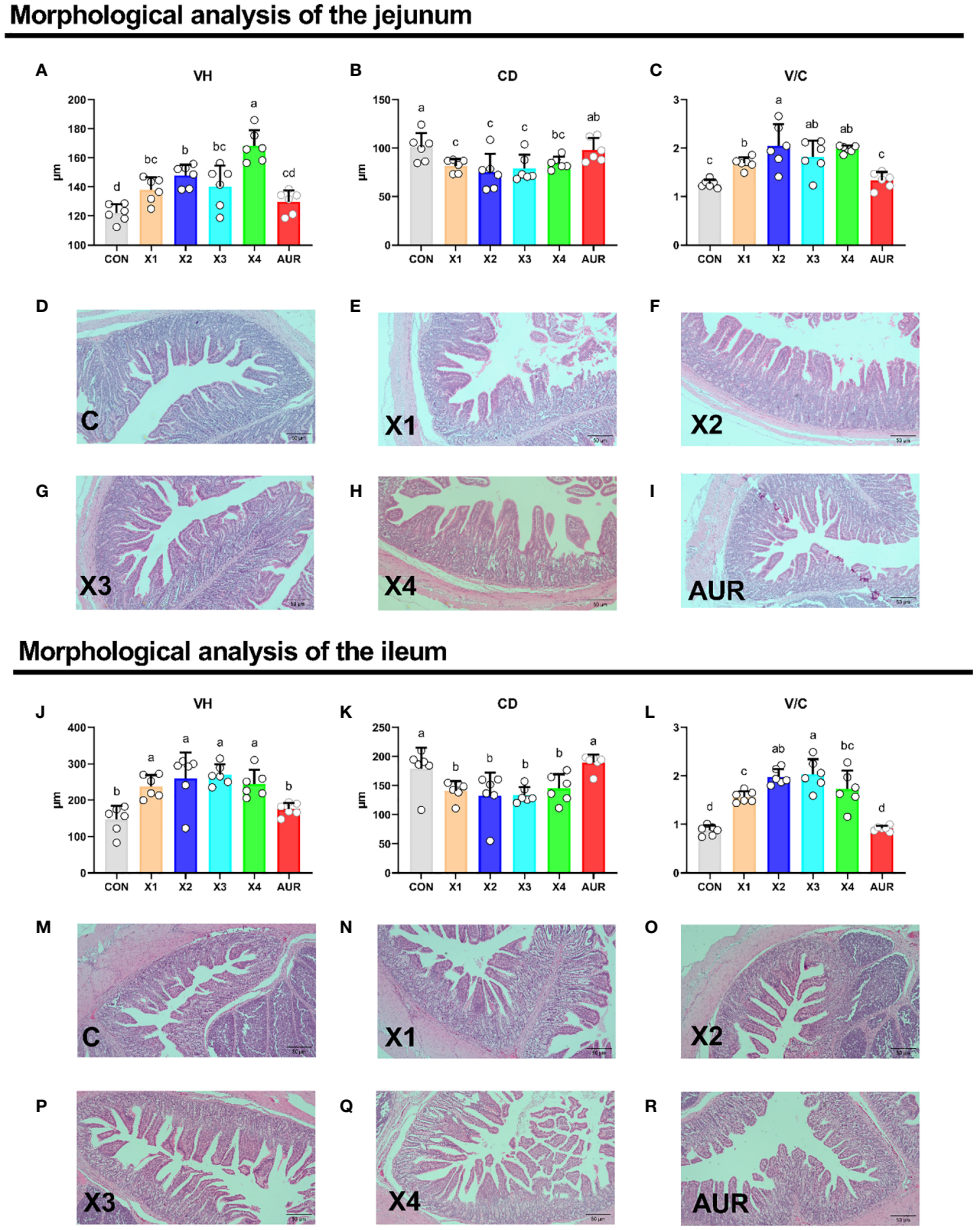
Effect of Xiasangju residues on intestinal morphology in weaned piglets. **(A–C)** are the villus height, crypt depth and villus height/crypt depth of jejunum tissue of weaned piglets, respectively. **(D–I)** shows HE stained sections of jejunal tissues with different treatments. **(J–L)** are the villus height, crypt depth and villus height/crypt depth of the ileum tissue of weaned piglets, respectively. **(M–R)** shows HE stained sections of different treatments of ileal tissues. CON, basal diet fed; AUR, 75 mg/kg chlortetracycline added to the basal diet; X1, 0.5% Xiasangju residues added to the basal diet; X2, 1.0% Xiasangju residues added to the basal diet; X3, 2.0% Xiasangju residues added to the basal diet; X4, 4.0% Xiasangju residues added to the basal diet; VH, villous height; CD, crypt depth; V/C, villous height/crypt depth. Means without the same superscript letter were significantly different at the *P*-value (n=6).

As shown in **Figure 2**, the addition of 0.5%, 1.0%, 2.0% and 4% Xiasangju residues to the diet of weaned piglets significantly increased VH and V/C and decreased CD in the jejunum and ileum of the piglets compared to the control group (*P* < 0.05).

### 3.7 Goblet cells

**Figure 3:**
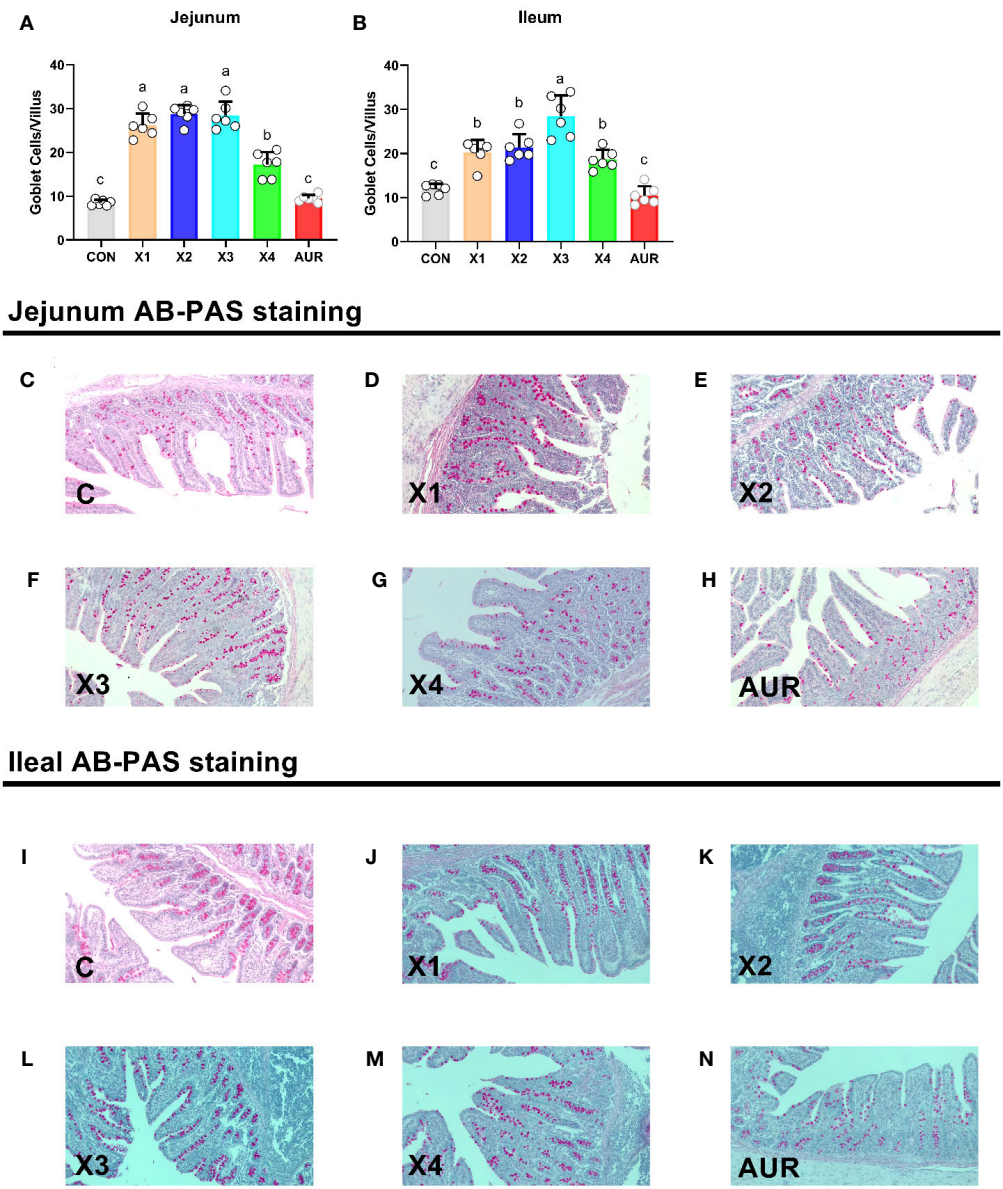
Effect of Xiasangju residues on intestinal goblet cells in weaned piglets. **(A, B)** are the number of villus goblet cells in jejunum and ileum of weaned piglets, respectively. **(C–H)** shows AB-PAS stained sections of jejunal tissues with different treatments. **(I–N)** are AB-PAS stained sections of ileum tissue from weaned piglets. CON, basal diet fed; AUR, 75 mg/kg chlortetracycline added to the basal diet; X1, 0.5% Xiasangju residues added to the basal diet; X2, 1.0% Xiasangju residues added to the basal diet; X3, 2.0% Xiasangju residues added to the basal diet; X4, 4.0% Xiasangju residues added to the basal diet. Means without the same superscript letter were significantly different at the *P*-value (n=6).

As shown in **Figure 3**, the addition of 0.5%, 1.0%, 2.0% and 4% Xiasangju residues to the diet of weaned piglets significantly increased goblet cells in the jejunum and ileum of the piglets compared to the control group (*P* < 0.05).

### 3.8 Expression of inflammation-related genes

**Figure 4:**
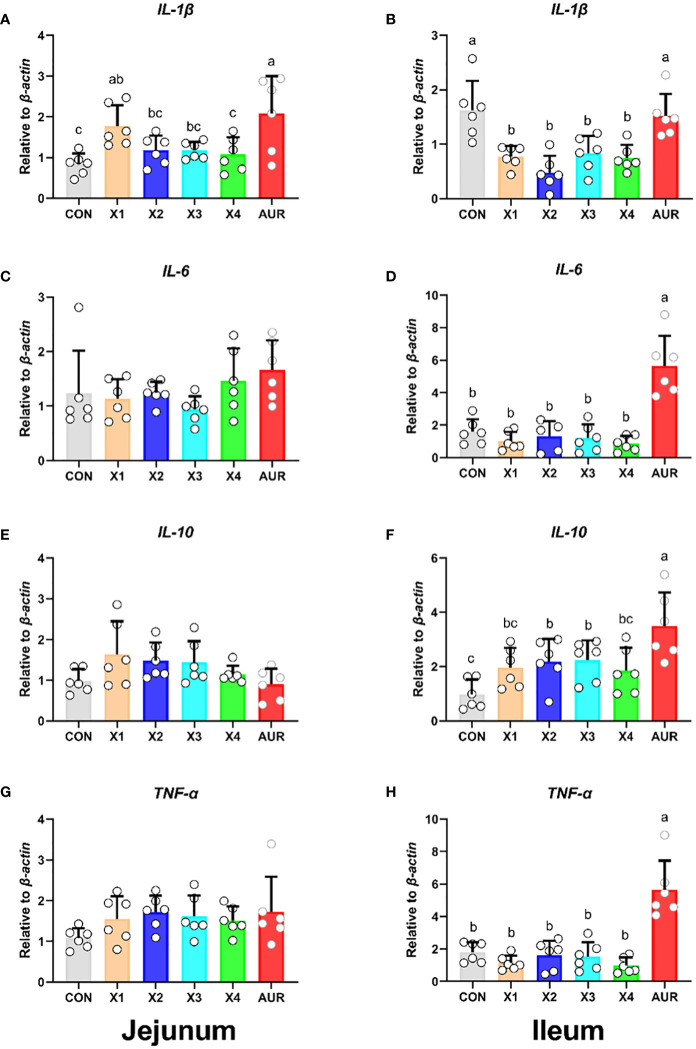
Effect of Xiasangju residues on the expression of intestinal inflammation-related genes in weaned piglets. **(A, C, E, G)** are the relative expression of *IL-1β, IL-6, IL-10* and *TNF-α* in jejunum tissue, respectively. **(B, D, F, H)** are the relative expression of *IL-1β, IL-6, IL-10* and *TNF-α* in ileum tissue, respectively. CON, basal diet fed; AUR, 75 mg/kg chlortetracycline added to the basal diet; X1, 0.5% Xiasangju residues added to the basal diet; X2, 1.0% Xiasangju residues added to the basal diet; X3, 2.0% Xiasangju residues added to the basal diet; X4, 4.0% Xiasangju residues added to the basal diet; *IL-1β*, interleukin-1 beta; *IL-6*, interleukin-6; *IL-10*, interleukin-10; *TNF-α*, tumor necrosis factor alpha. Means without the same superscript letter were significantly different at the *P*-value (n=6).

As shown in **Figure 4**, 0.5% Xiasangju residues significantly increased jejunal *IL-1β* relative expression compared with the control group (*P* < 0.05), although Xiasangju residues had no significant effect on jejunal *IL-6*, *IL-10* and *TNF-α* relative expression (*P* > 0.05). Interestingly, 0.5%, 1.0%, 2.0%, and 4.0% Xiasangju residues significantly decreased ileal *IL-1β* relative expression, and 1.0% and 2.0% Xiasangju residues significantly increased *IL-10* relative expression (*P* < 0.05), with nonsignificant effects on *IL-6* and *TNF-α* (*P* > 0.05).

### 3.9 Expression of intestinal barrier-related genes

**Figure 5:**
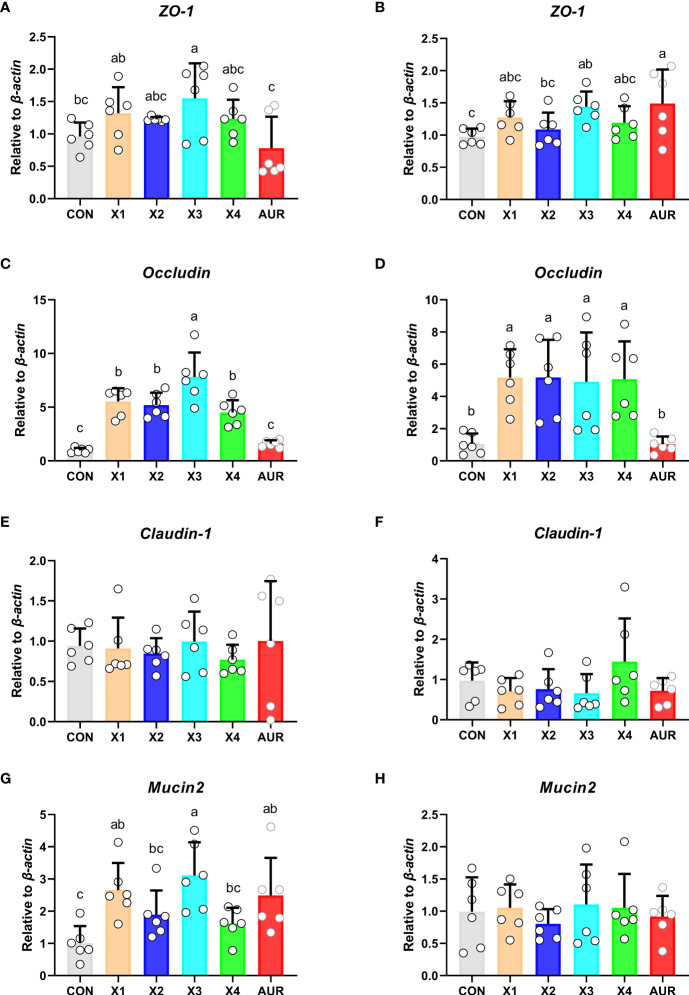
Effect of Xiasangju residues on the expression of genes related to intestinal barrier in weaned piglets. **(A, C, E, G)** are the relative expressions of *ZO-1, Occludin, Claudin-1* and *Mucin2* in jejunum tissue, respectively. **(B, D, F, H)** are the relative expressions of *ZO-1, Occludin, Claudin-1* and *Mucin2* in ileum tissue, respectively. CON, basal diet fed; AUR, 75 mg/kg chlortetracycline added to the basal diet; X1, 0.5% Xiasangju residues added to the basal diet; X2, 1.0% Xiasangju residues added to the basal diet; X3, 2.0% Xiasangju residues added to the basal diet; X4, 4.0% Xiasangju residues added to the basal diet; *ZO-1*, tight junction protein 1. Means without the same superscript letter were significantly different at the *P*-value (n=6).

Compared with the control group, treatment with 2% significantly increased the relative expression of *ZO-1* in the jejunum and ileum (**Figures 5A, B**) (*P* < 0.05), treatment with 0.5%, 1%, 2.0%, and 4% significantly increased the relative expression of *Occludin* in the jejunum and ileum (**Figures 5C, D**) (*P* < 0.05), and treatment with 0.5% and 2% significantly increased the relative expression of *Mucin2* in the jejunum (**Figure 5G**) (*P* < 0.05). *Claudin-1* in the jejunum and ileum (**Figures 5E, F**) and *Mucin2* in the ileum (**Figure 5H**) did not have a significant effect (*P* > 0.05).

### 3.10 Colonic bacterial richness, diversity, and similarity

**Figure 6:**
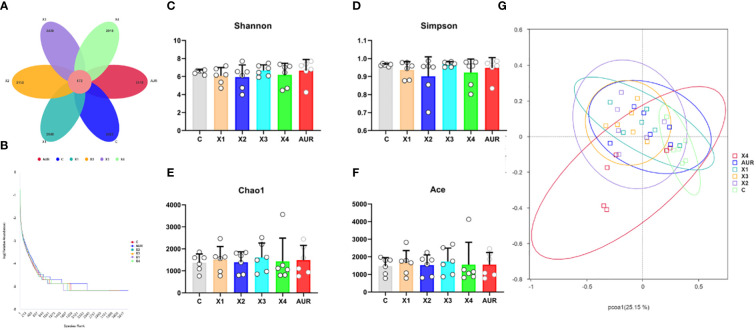
Abundance, diversity and similarity of colonic microbiota (n=6). **(A)**, petal plot of the number of common and uniquely observed taxonomic units (OTUs) in the colonic contents of weaned piglets on day 21 of the experiment. **(B)**, Rank Abundance curves of gut microbiota in the colonic contents of weaned piglets on day 21 of the experiment. **(C–F)** are the Shannon index, Simpson index, Chao1 index, and ACE index of the colonic microbiota of weaned piglets on day 21 of the experiment, respectively. **(G)** shows principal coordinate analysis (PCoA) plot of the gut microbiota in the colonic contents of weaned piglets on day 21 of the experiment. CON, basal diet fed; AUR, 75 mg/kg chlortetracycline added to the basal diet; X1, 0.5% Xiasangju residues added to the basal diet; X2, 1.0% Xiasangju residues added to the basal diet; X3, 2.0% Xiasangju residues added to the basal diet; X4, 4.0% Xiasangju residues added to the basal diet.

​To investigate the effect of dietary therapy on the bacterial community of the piglets, 16S rRNA gene sequencing was performed on a colonic digested sample of the piglets on day 21 of the trial (**Figure 6**). A total of 13951 operational taxonomic units (OTUs) were identified in chyme of the colon, with a total of 672 OTUs. Compared to the control group, the unique OTUs in the Xiasangju residues treatment group initially increased with increasing concentration and then decreased at 4%. There were 405 more unique OTUs in the 3% Xiasangju residue treatment group than in the control group. Alpha diversity is a basic indicator for evaluating bacterial richness and diversity, and Shannon, Simpson, Chao1, and Ace indices were not significantly different among treatments in this experiment. Subsequently, Bray−Curtis was used to analyze the β diversity of the microbiota in the colonic chyme of piglets, and no significant difference was found among all treatments.

### 3.11 Colonic microbiota composition

**Figure 7:**
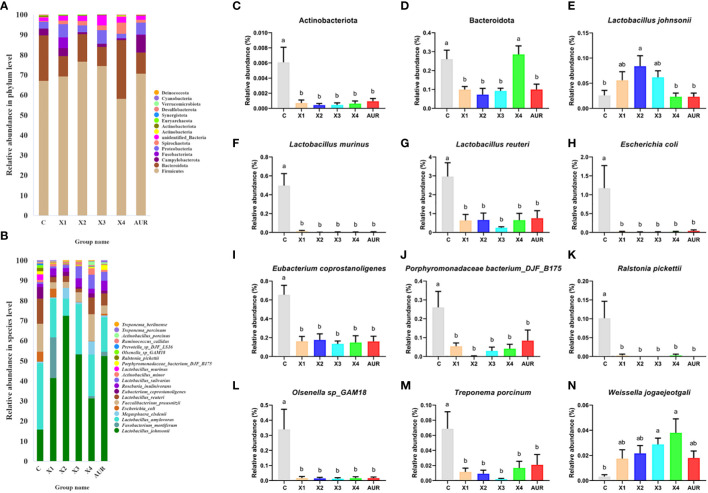
Structure of colon microbiota (n=6). **(A, B)** shows the TOP20 species composition at the phylum level and species level of the piglet colon, respectively. **(C–N)** represents the relative abundance of Actinobacteriota, Bacteroidota, *Lactobacillus johnsonii, Lactobacillus murinus, Lactobacillus reuteri, Escherichia coli, Eubacterium coprostanoligenes, Porphyromonadaceae bacterium_DJF_B175, Ralstonia pickettii, Olsenella sp_GAM18, Treponema porcinum* and *Weissella jogaeotgali*, respectively. CON, basal diet fed; AUR, 75 mg/kg chlortetracycline added to the basal diet; X1, 0.5% Xiasangju residues added to the basal diet; X2, 1.0% Xiasangju residues added to the basal diet; X3, 2.0% Xiasangju residues added to the basal diet; X4, 4.0% Xiasangju residues added to the basal diet. Means without the same superscript letter were significantly different at the *P*-value (n=6).

Next, we assessed the colonic microbiota composition of weaned piglets at both the phylum and species levels (**Figure 7A**). At the phylum level, Firmicutes, Bacteroidota, Campylobacterota, Fusobacteriota, Proteobacteria, Spirochaetota, unidentified_Bacteria, Actinobacteria, Actinobacteriota and Euryarchaeota were the main components of the colonic microbiota. Notably, Actinobacteriota and Bacteroidota were significantly decreased by 0.5%, 1.0%, 2.0%, and 4% Xiasangju residues and 0.5%, 1.0%, and 2.0% Xiasangju residues, respectively, compared with the control (*P* < 0.05).

At the species level, the Xiasangju residues significantly altered the colonic microbiota structure of the piglets (**Figure 7B**). The 1% Xiasangju residues significantly increased the abundance of *Lactobacillus johnsonii*, and the 2% and 4% Xiasangju residues significantly increased the abundance of *Weissella jogaeotgali* compared to the control group (*P* < 0.05). Interestingly, the abundance of *Lactobacillus murinus*, *Lactobacillus reuteri*, *Escherichia coli*, *Eubacterium coprostanoligenes*, *Porphyromonadaceae bacterium_DJF_B175*, *Ralstonia pickettii*, *Olsenella sp_GAM18*, and *Treponema porcinum* was significantly reduced in all treatments (*P* < 0.05).

## 4 Discussion

Intracellular damage and the proliferation of pathogenic bacteria are often induced by changes in the feed, physiology, psychology and environment of piglets after weaning, resulting in diarrhea and reduced growth performance of piglets, resulting in significant economic losses in pig breeding (Li et al., 2021; Tang et al., 2022). ​In the current context of antibiotic-free pig diets and treatment with few antibiotics, it is particularly important to find nutritional regulation measures to alleviate the stress of weaning in piglets. Previous studies have shown that adding Chinese herbal medicines to feed improves the growth performance of piglets, increases the feed-to-gain ratio, and reduces diarrhea rates (Canibe et al., 2022). In this study, we found that the residues of Xiasangju contain high contents of linoside, rosmarinic acid, isogrosmarinic acid, acacetin, caffeic acid, chlorogenic acid, neochlorogenic acid, cryptochlorogenic acid, isochlorogenic acid A, isochlorogenic acid B and isochlorogenic acid C. Most of these substances have anti-inflammatory, antibacterial and antioxidant effects. However, there is no report on the application of Xiasangju residues in livestock and poultry production. In the present trial, we observed that supplementation with 0.5%, 1.0%, and 2.0% of the Xiasangju residues significantly reduced the diarrhea score of the piglets, similar to the results of chlortetracycline. ​After weaning, piglets are prone to diarrhea due to changes in dietary status and environment, their own digestive systems are not fully developed, their resistance is weak, and the condition can even be life threatening (Jayaraman and Nyachoti, 2017; Gao et al., 2019). In the present study, supplementation of weaned piglets with Xiasangju residues significantly reduced the diarrhea score of the piglets. The reduction in diarrhea in weanling piglets caused by Xiasangju residues may be due to the presence of lutein, caffeic acid, and chlorogenic acid, which inhibit pathogenic bacteria (Köksal et al., 2017; Sun et al., 2022; Zhang et al., 2022), are anti-inflammatory and improve the intestinal barrier (Naveed et al., 2018; Kim et al., 2023), as also confirmed in this study.

Serum biochemical parameters are important indicators for the clinical assessment of animal health and metabolic status. AST, ALP, and LDH are intracellular enzymes that are released into the blood when cells are damaged (Zaitsev et al., 2021). In the present study, serum AST, ALP, and LDH were significantly reduced by the addition of Xiasangju residues, suggesting that Xiasangju residues are beneficial for liver and cardiac health in weaned piglets. At the same time, Xiasangju residues also improved ALB, BUN, GLU, and LDL-C, suggesting that Xiasangju residues may be beneficial for glucose and lipid metabolism and protein metabolism in piglets. Chen et al. also observed an increase in serum ALB and a decrease in BUN in weaned piglets supplemented with chlorogenic acid, similar to the results of the present study (Chen et al., 2018a). They are the main antibodies involved in the body’s immune response. IgA, IgG and IgM are the main immunoglobulins in animal serum, of which IgG and IgM help the body defend against the invasion of bacteria and viruses, and IgA has the immunological activity of IgG and IgM (Castro-Dopico and Clatworthy, 2019; Perše and Večerić-Haler, 2019). In this study, we observed that the presence of heparin residue increases the serum IgM and IgG contents of piglets, suggesting that heparin residue may improve the immune capacity of piglets. Some studies have also observed an immune-boosting effect of Xiasangju residues in chickens, rabbits, and fish (Chen et al., 2022; Jin et al., 2022; Liu et al., 2022). Secretory immunoglobulin A (sIgA) is an important antibody in the intestinal tract. It recognizes lipopolysaccharides, capsular polysaccharides, and flagellins on the surface of some bacteria, forms sIgA to coat the bacteria, prevents pathogens from contacting intestinal epithelial cells, enhances intestinal barrier function, reduces the pathogen proinflammatory response, and thus protects the intestinal tract (Ding et al., 2021; Seikrit and Pabst, 2021). In the present study, supplementation of the diet of weanling piglets with 1%, 2% and 4% Xiasangju residues significantly increased the sIgA content of ileus digesta, suggesting that Xiasangju residues are beneficial to the immunity of the mucosal lining of the gut of weanling piglets.

The intestine is the main site of nutrient digestion and absorption and is composed of villi and crypts covered by a single layer of columnar epithelial cells. Changes in its structure directly affect the digestion and absorption of nutrients. However, early weaning stress can damage the gut structure of the piglet, mainly by reducing the height of the villus and deepening the crypt depth (Cao et al., 2022; Tang et al., 2022). In this experiment, supplementation with Xiasangju residues significantly increased the villous height of the jejunum and ileum, significantly decreased the crypt depth, and significantly increased the ratio of villous height to crypt depth, suggesting that Xiasangju residues are beneficial in improving intestinal structural damage in weanling piglets. Chlorogenic acid, which is the main active ingredient in Xiasangju residues, has been reported to increase the ratio of villus height and crypt depth in the small intestine of piglets and to decrease crypt depth (Zhang et al., 2018; Qin et al., 2023). Goblet cells are the most abundant secretory cells in the epithelium of the gut and mainly secrete trefoil factor and mucin 2. They are essential for clearing the gut epithelium of external pathogens and allergens, so they protect against many potential threats (Birchenough et al., 2015; Gustafsson and Johansson, 2022). In the present study, Xiasangju residues significantly increased the number of goblet cells in the jejunum and ileum villi of weanling piglets, suggesting that Xiasangju residues are beneficial for the proliferation of goblet cells (Qin et al., 2023).

The mechanical barrier is the first line of defense to prevent harmful substances from invading the intestinal mucosa and disrupting intestinal homeostasis. The mechanical barrier consists mainly of tight junctions between the epithelium and cells of the gut mucosa, mainly containing claudin, occludin, and zonula occludens, which are the main determinants of the mechanical barrier (Okumura and Takeda, 2017; Sylvestre et al., 2023). Previous studies have shown that traditional Chinese medicine and the plant extract cacao can improve the mechanical barrier and maintain intestinal barrier function by regulating intestinal mucosal epithelial cells and intercellular junctions, increasing intestinal mucosal blood flow, and promoting intestinal peristalsis (Che et al., 2022; Wang et al., 2022). In this study, supplementation of Xiasangju residues to the diet of weanling piglets increased the relative mRNA expression of *ZO-1* and *Occludin* in the jejunum and ileum, and jejunum *Mucin2* was also significantly increased when Xiasangju residues were added to the diet of weanling piglets, indicating that Xiasangju residues were beneficial to the intestinal barrier function of piglets. Cytokines also play an important role in the regulation of the intestinal tight junction barrier (Chen et al., 2021). Previous studies have shown that the active ingredient in Xiasangju residues has a good anti-inflammatory effect, increasing the expression and secretion of anti-inflammatory factors such as IL-10 and decreasing the expression and secretion of pro-inflammatory factors (Chen et al., 2018b; Formiga et al., 2020; Zielińska et al., 2021). In this study, we also observed that Xiasangju residues could increase the relative expression of *IL-10* mRNA and decrease the relative expression of *IL-1β* mRNA in the ileum, indicating that Xiasangju residues could improve the inflammatory state of weanling piglets.

The gut microbiome plays an important role in the digestion and absorption of nutrients and in animal health. Early weaning can lead to a disturbed gut flora in piglets, and rapid proliferation of intestinal pathogens such as *Escherichia coli* can cause weaning diarrhea (Gresse et al., 2017; Guevarra et al., 2019). In this study, while the alpha and beta diversity of the colonic microbiome of *Weissella jogaeotgali* and *Lactobacillus johnsonii* was not significantly affected at the species level, the relative abundance of two typical probiotics, *Lactobacillus johnsonii* and *Weissella jogaeotgali*, was increased at the species level. At the same time, it reduced the relative abundance of *Escherichia coli* and *Treponema porcinum*, indicating that Xiasangju residues can improve the gut microbiota of weaned piglets, which may be one of the main reasons that Xiasangju residues improve diarrhea and the intestinal barrier of piglets, but further verification is needed. The dietary fiber and active ingredients in the residue may be beneficial for the growth and proliferation of *Lactobacillus johnsonii* and *Weissella jogaeotgali*, among others. At the same time, the growth of pathogenic bacteria is inhibited (Makki et al., 2018; Chen et al., 2019; Wang et al., 2023). *Lactobacillus johnsonii* and *Weissella jogaeotgali* are two important probiotics that produce acid, inhibit bacteria and have good adhesion to intestinal epithelial cells. It performs well in improving the structure of the gut microbiota and enhancing the integrity of the intestinal barrier (Teixeira et al., 2021; Bai et al., 2022; Han et al., 2022; Fanelli et al., 2023).

## 5 Conclusion

In summary, dietary supplementation with Xiasangju residues alleviates diarrhea caused by early weaning, improves intestinal barrier and immune status, and modulates gut microbiota composition, suggesting that Xiasangju residues have some potential as an antibiotic alternative. However, this study was only a preliminary study and the Xiasanju residues did not have a significant effect on the weight gain and feed/gain of the piglets. Large groups and long-term feeding trials are necessary in the future.

## Data availability statement

The datasets presented in this study can be found in online repositories. The names of the repository/repositories and accession number(s) can be found below: NCBI, PRJNA1011898.

## Ethics statement

The animal study was approved by Hunan Agricultural University Institutional Animal Care and Use Committee (202105). The study was conducted in accordance with the local legislation and institutional requirements.

## Author contributions

WS: Data curation, Formal Analysis, Methodology, Project administration, Writing – original draft, Writing – review & editing. ZC: Data curation, Formal Analysis, Methodology, Project administration, Writing – original draft, Writing – review & editing. ZH: Data curation, Formal Analysis, Software, Writing – review & editing. AW: Data curation, Writing – review & editing. MZ: Conceptualization, Methodology, Project administration, Resources, Supervision, Validation, Visualization, Writing – review & editing. JG: Writing – review & editing.
